# Low-frequency parietal repetitive transcranial magnetic stimulation reduces fear and anxiety

**DOI:** 10.1038/s41398-020-0751-8

**Published:** 2020-02-17

**Authors:** Nicholas L. Balderston, Emily M. Beydler, Madeline Goodwin, Zhi-De Deng, Thomas Radman, Bruce Luber, Sarah H. Lisanby, Monique Ernst, Christian Grillon

**Affiliations:** 1 Section on Neurobiology of Fear and Anxiety, National Institute of Mental Health, National Institutes of Health Bethesda, Bethesda, MD USA; 2 Center for Neuromodulation in Depression and Stress, Department of Psychiatry, University of Pennsylvania Philadelphia, Philadelphia, PA USA; 3 Noninvasive Neuromodulation Unit, National Institute of Mental Health, National Institutes of Health Bethesda, Bethesda, MD USA

**Keywords:** Human behaviour, Psychiatric disorders

## Abstract

Anxiety disorders are the most prevalent mental disorders, with few effective neuropharmacological treatments, making treatments development critical. While noninvasive neuromodulation can successfully treat depression, few treatment targets have been identified specifically for anxiety disorders. Previously, we showed that shock threat increases excitability and connectivity of the intraparietal sulcus (IPS). Here we tested the hypothesis that inhibitory repetitive transcranial magnetic stimulation (rTMS) targeting this region would reduce induced anxiety. Subjects were exposed to neutral, predictable, and unpredictable shock threat, while receiving double-blinded, 1 Hz active or sham IPS rTMS. We used global brain connectivity and electric-field modelling to define the single-subject targets. We assessed subjective anxiety with online ratings and physiological arousal with the startle reflex. Startle stimuli (103 dB white noise) probed fear and anxiety during the predictable (fear-potentiated startle, FPS) and unpredictable (anxiety-potentiated startle, APS) conditions. Active rTMS reduced both FPS and APS relative to both the sham and no stimulation conditions. However, the online anxiety ratings showed no difference between the stimulation conditions. These results were not dependent on the laterality of the stimulation, or the subjects’ perception of the stimulation (i.e. active vs. sham). Results suggest that reducing IPS excitability during shock threat is sufficient to reduce physiological arousal related to both fear and anxiety, and are consistent with our previous research showing hyperexcitability in this region during threat. By extension, these results suggest that 1 Hz parietal stimulation may be an effective treatment for clinical anxiety, warranting future work in anxiety patients.

## Background

Anxiety disorders are the most commonly diagnosed class of mental disorders. Nearly 20% of the US population meets the criteria for an anxiety disorder within a given year, and less than half of those individuals receive minimally adequate treatment for their disorder^1^. One potential reason for this lack of treatment efficacy is that clinical anxiety is comprised of an array of complex symptoms involving cognitive and behavioral domains^2^, however, much of the mechanistic anxiety research remains focused on a narrow set of subcortical regions, such as the amygdala and the bed nucleus of the stria terminalis^3–5^. Therefore, in order to better understand and treat clinical anxiety, it is important to broaden the scope of the research into anxiety mechanisms, and develop treatment options specifically targeted at these mechanisms.

One avenue for novel treatments is transcranial magnetic stimulation (TMS),which is a is a noninvasive neuromodulation approach that uses brief magnetic pulses generated at the scalp to induce electrical currents in the underlying cortical neurons^6^. Repetitive TMS (rTMS), which was recently approved by the FDA to treat depression^7^, uses repeated stimulation of a specific region to induce long-lasting changes in cortical excitability^8^. In motor cortex, high-frequency stimulation (>5 Hz) increases cortical excitability, while low-frequency stimulation decreases cortical excitability^9–11^. Using this approach it is possible to target specific regions of the cortex with high spatial and temporal accuracy.

Previous studies using electroencephalography (EEG) have demonstrated hyperactivity in the parietal cortex as a function of arousal in anxious patients^12^, suggesting a potential link between parietal hyperactivity and attention control deficits^13^. Consistent with these results, our previous work used unbiased data-driven approaches in a multimodal (MEG/fMRI) neuroimaging study of effects of threat-of-shock-induced anxiety on cortical excitability (alpha desynchronization) and global brain connectivity^14^. In that study, we found evidence for increases in both excitability and functional connectivity of the intraparietal sulcus in anxious subjects anticipating the shock.

Based on these results, we hypothesized that the parietal cortex may be an effective target for noninvasive neuromodulation. In this study, we targeted the IPS with low-frequency rTMS to reduce parietal hyperexcitability during anxiety. We then measured the effect of this stimulation protocol on fear- and anxiety-potentiated startle during the Neutral, Predictable, and Unpredictable threat task (NPU), which explores defense responses to predictable and unpredictable threat^15,16^. We hypothesized that low-frequency stimulation of the parietal cortex would decrease the heightened orienting to the white noise threat, and that this would attenuate potentiated startle observed during the threat periods.

## Materials and methods

### Participants

Sample size was based on our previous study exploring the effect of right dlPFC rTMS on anxiety, which used a similar design^17^. Twenty-five participants were enrolled in the study. All subjects met the inclusion/exclusion criteria, which included: aged 18–50, English speaking, no Axis I diagnosis^18^, no medication use, no neurological issues, and no MRI/TMS contraindications. Of the enrolled subjects, seven were withdrawn from the study for the following reasons: one subject was unable to attend all study sessions, one subject was not compliant during the TMS procedure, one subject had neurological condition revealed on the MRI scan, the equipment failed for one subject, we were unable to find the motor threshold for one subject, and one subject had an abnormally low percentage of startle responses to the white noise presentations (<30%). The remaining 19 subjects (mean age = 29.11 years, SD = 8.47), included 13 females. All participants gave written informed consent approved by the National Institute of Mental Health (NIMH) Combined Neuroscience Institutional Review Board and were compensated for their time.

### NPU procedure

#### NPU threat task

**Fig. 1:**
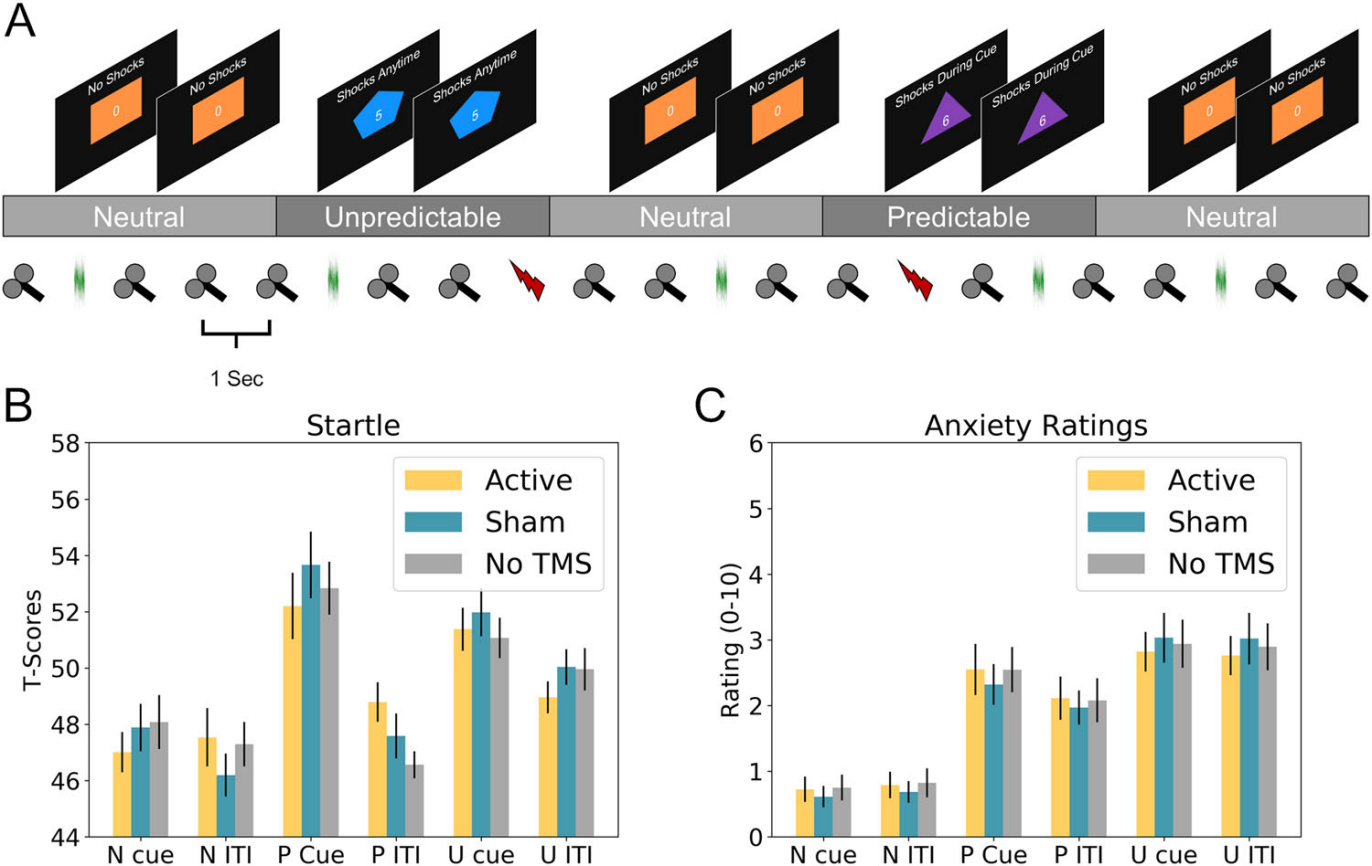
Design Schematic and overall results. **a** Schematic of the neutral predictable unpredictable (NPU) threat task with online 1 Hz repetitive transcranial magnetic stimulation (rTMS). Subjects were exposed to neutral (N), predictable (P), and unpredictable (U) conditions. During the entire run subjects receive 1 Hz active rTMS, sham rTMS, or no TMS (tones). Startle probes and shocks were embedded in this 1 Hz train at random intervals during the cue (shape) period or inter trial interval (ITI). **b** Startle responses during the cue and ITI periods of the NPU task as a function of rTMS type. **c** Concurrent anxiety ratings collected during the NPU task and sampled during the cue and ITI periods as a function of rTMS type. TMS coil icons represent TMS pulses. Green noise traces represent white noise presentations. Lightning bolts represent shocks. Bars represent mean ± standard error.

We used the neutral, predictable, unpredictable threat task to induce fear and anxiety (see Fig. 1). The NPU task consisted of three runs^15^. Within each run, there were several blocks of neutral (no shock), predictable (at risk for shock only during cue), and unpredictable blocks (at risk for shock at all times). Within each block, there were several trials where a cue (shape) was presented for 8 s. Cues were simple colored (orange, teal, purple) geometric shapes (triangle, square, pentagon), with color and shape randomly assigned to conditions. White noise probes were presented during each cue presentation, and during an equal number of trial interval (ITI) periods. White noise probes were presented every ~17 ± 4 s. Three shocks were presented in each run at a random point during either the cue (predictable condition) or the ITI (unpredictable condition). To measure anxiety, we recorded the amplitude of the blink response elicited by the white noise. We also measured anxiety using a concurrent continuous rating scale. The three runs differed based on the stimulation type. During one run, subjects received 1 Hz active rTMS. During another, they received 1 Hz sham rTMS. During a third, they received 1 Hz tone presentations (a “no TMS” control). The order of the runs were counterbalanced across subjects, and both the operator and the subject were blinded to the type of stimulation (active vs. sham) during the active and sham runs. White noises and shocks were embedded in the TMS/tone series by replacing the TMS pulse (or tone) at random points during the blocks.

#### White noise

The startle stimulus was a 40-ms, 103-dB white noise with an instantaneous rise time^19^. Because the white noise needed to be delivered during the rTMS, subjects required headphones with hearing protection. Therefore, subjects wore custom headphones fabricated from noise-cancelling ear muffs with a noise reduction rating of 30 dB (3 M Optime 105; Minneapolis, MN). Prior to the runs, subjects were exposed to nine unsignaled presentations of the white noise with a variable inter-noise interval of ~17 s to reduce initial startle reactivity.

#### Shock

The shock was a 100 ms, 200 Hz train of stimulation delivered to the right wrist via 2, 11 mm disposable Ag/AgCl electrodes (Biopac Item number EL508; Goleta, CA), spaced ~2 cm apart using a constant current stimulator (Digitimer #DS7A, Ft. Lauderdale, FL). Shock intensity (*M* = 3.74 mA; SD = 3.07 mA) was set to a level that subjects rated as “uncomfortable but tolerable”.

#### Electromyography

Facial electromyography (EMG) startle responses were recorded from the left orbicularis oculi muscle via 15 × 20 mm hydrogel coated vinyl electrodes (Rhythmlink #DECUS10026; Columbia, SC), and the EMG signal was sampled at 2000 Hz using a Biopac MP160 unit (Biopac; Goleta, CA).

#### Startle measure

The EMG signal was filtered from 30 to 300 Hz, then rectified and smoothed using a 20-ms sliding window. Responses were scored as the peak (20–120 ms)—the baseline (−50 to 0 ms), and converted to *t*-scores (*t*_x_ = [*Z*_x_ × 10] + 50). Trials with excessive noise (baseline SD > 2x run SD) were counted as missing data, and trials with no discernible blink (peak < baseline voltage amplitude range) were coded as 0.

#### Anxiety ratings

Subjects rated their anxiety throughout the experiment using keypresses that updated an online rating scale (from 0 [not anxious] to 10 [extremely anxious]). This was sampled at each white noise presentation, and averaged across trials.

### rTMS procedure

#### Motor threshold determination

Resting motor threshold (MT) was measured in the first dorsal interosseous (FDI) muscle, and was defined as the minimum magnetic flux needed to elicit a threshold motor evoked potential (MEP) ≥ 50 µV in 5 out of 10 trials^20,21^. Subjects MT averaged 44.63 (SD = 8.84) percent of machine output.

#### rTMS

Subjects received 1 Hz rTMS to either the left or right intraparietal sulcus (defined below) via a Cool-B65 A/P coil powered by a MagVenture MagPro 100 (MagVenture, Inc., Alpharetta GA) stimulator. During the active run, subjects received pulses at 100% of MT for the duration of the ~870 s run. During the sham run, subjects received the same number of pulses from the unmarked placebo side of the coil, which provides a field reduction of ~80%. Both the subject and the operator were blinded to the condition (active vs. sham). Immediately after the experiment, subjects were asked to guess which run was active and provide a confidence rating (1 [not sure] to 10 [very sure]) after the experiment.

### Target localization

**Fig. 2:**
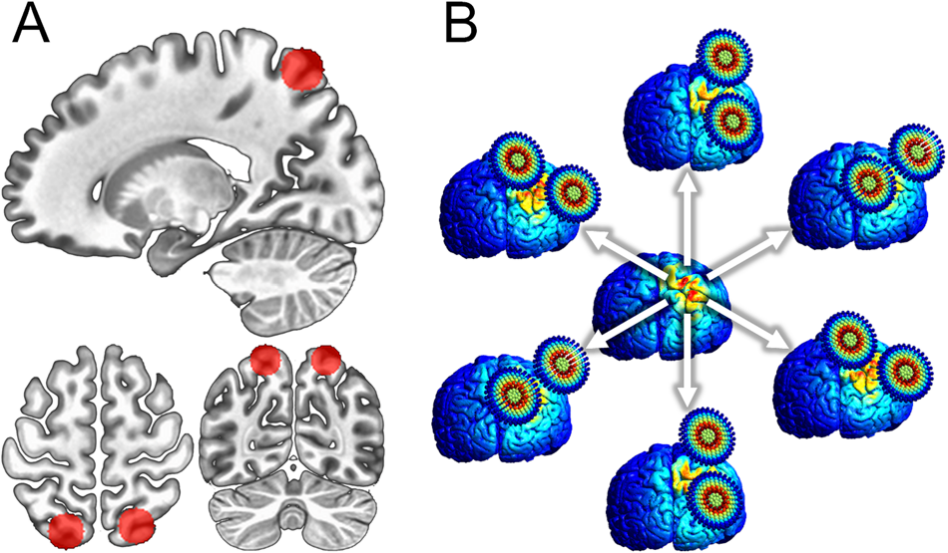
Schematic of targeting and electric- (e) field modelling methods. **a** 10 mm spheres drawn around peak global brain connectivity coordinates from Balderston et al. ^14,16^, overlaid on MNI template Red circles represent search region for individual targets, which were defined at the individual subject level as the voxel within the mask with the highest global brain connectivity. **b** Representation of e-field modelling approach used in the current analysis. Multiple e-field models corresponding to equally spaced coil orientations were conducted. The coil orientation that yielded the e-field model with the maximum value at the target was used during the TMS session.

We based our target choice on group data from Balderston et al. (see Fig. 2a)^14,16^, which found increases in global connectivity in the intraparietal sulcus during threat-of-shock, suggesting that this region may be a connectivity hub mediating anxiety expression. Accordingly, the goal of the present work was to extract the location within this region with the highest global connectivity. We then used electric-field modelling to optimize coil placement (See Fig. 2b), and online neuronavigation to ensure accurate stimulation.

#### MRI scans

For high-resolution structural imaging and to calculate the e-field models, we collected a T1 (Resolution = 0.8 mm; FOV = 256 × 256; Slices = 176 sagittal; TR = 2400 ms; TE = 2.24 ms; Flip angle = 7°), a T2 (Resolution = 0.8 mm; FOV = 300 × 320; Slices = 208 sagittal; TR = 3200 ms; TE = 566 ms; Flip angle = 120°), and a DWI (Resolution = 2 mm; FOV = 128 × 128; Slices = 70 axial; TR = 12000 ms; TE = 64 ms; Flip angle = 90°; B0 = 100; Directions = 30) scan. To calculate global connectivity, we collected a 10 min eyes-open, multi-echo, resting-state EPI (Resolution = 3 mm; FOV = 64 × 64; Slices = 32 axial; TR = 2000 ms; TEs = 13.8, 31.2, 48.6 ms; Flip angle = 70°) scan.

#### MRI/fMRI processing

Reconstruction and fMRI pre-processing was done with AFNI^22,23^. fMRI preprocessing included slice-timing correction, despiking, volume registration, TE-dependent independent components analysis (ICA) denoising, scaling, EPI distortion correction, motion scrubbing, and blurring with a 6 mm FWHM Gaussian kernel. Timeseries were further denoised based on regressors of no interest corresponding to the 6 motion parameters and 4 polynomial baseline estimates. MRI and DWI processing was done using the SimNIBS^24^ software package, which calls Freesurfer to create tissue compartments from the T1/T2 images for the skin, skull, cerebrospinal fluid, grey matter, and white matter, and FSL to compute conductivity tensors from the DWI images.

#### fMRI-guided target selection

To localize the optimal IPS target, we drew 10 mm spheres around the MNI coordinates reported in Balderston et al.^14,16^. We then calculated global connectivity from the denoised resting-state EPI scan using the AFNI tool 3dTCorrMap. Finally, we extracted the voxel with the highest global connectivity, and used the coordinates as the TMS target. Importantly, because the Balderston et al.^14,16^ finding was bilateral, we did not constrain our search to a specific hemisphere. Therefore, we had roughly equal numbers of people receiving left (*N* = 9) and right (*N* = 10) IPS stimulation.

#### Electric-field optimization

Once the target coordinates were identified, they were projected to the scalp, and a simulated coil was placed tangentially to the scalp surface. We then computed a series of 24 independent electric-field models corresponding to coil positions with equally spaced yaw vectors around the target^25^. The simulation with the largest normalized electric-field strength estimate at the original coordinates was used to define the yaw vector (coil orientation) during stimulation.

#### Neuronavigation

Prior to stimulation, subjects were registered to their T1 image and target via fiducial points at the nasion and tragi. During stimulation, the relative position of the subject and coil were tracked in real-time using Brainsight (Rogue Research Inc., Montreal, Canada), a frameless stereotaxic neuronavigation system that uses reflective markers monitored with an infrared camera.

## Results

### Blinding

To gauge the effectiveness of our blinding procedure, we tabulated the number of subjects who correctly guessed which run was active vs. sham. Subjects correctly guessed the active run at a rate of 76.47%, which is above chance (13 of 17 with missing data for 2 subjects; *Χ*^2^ (1, *N* = 17) = 4.77, *p* = 0.029). We also calculated the confidence in these guesses, which was not significantly different from the middle point of the confidence scale (*M* = 6.71; SD = 2.82; *t*(17) = 1.71, *p* = 0.107). Given that subjects were able to correctly guess the active run at an above chance level, we took extra steps below to rule out any placebo effects in the analyses.

### Startle

**Fig. 3:**
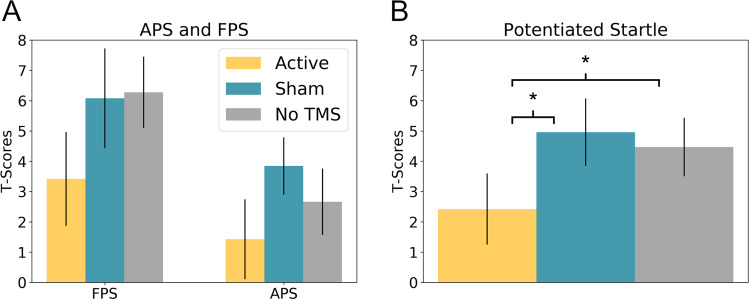
Fear and anxiety-potentiated startle results. **a** Fear and anxiety-potentiated startle (FPS and APS, respectively) as a function of repetitive transcranial magnetic stimulation (rTMS) type. **b** Potentiated startle (average of FPS and APS) as a function of rTMS type. Active 1 Hz rTMS to the parietal cortex significantly reduces potentiated startle compared to both sham and no TMS conditions. Bars represent mean ± standard error. *p > 0.05.

To quantify fear and anxiety, we calculated fear- and anxiety-potentiated startle (Fig. 3)^15,16^. For FPS, we subtracted the startle magnitude during the predictable ITI from the startle magnitude during the predictable cue. For APS, we subtracted the startle magnitude during the neutral ITI from the startle magnitude during the unpredictable ITI. We then performed a 2 (Startle type: FPS vs. APS) × 3 (Stimulation type: Active, vs. Sham, vs. No TMS) repeated measures ANOVA on the results. We found a main effect for Startle type with FPS being significantly larger than APS (*f*(1,18) = 8.05; *p* = 0.011), as well as a significant main effect for TMS (*f*(2,36) = 3.33; *p* = 0.047), but no interaction (*f*(2,36) = 0.36; *p* = 0.701).

To characterize the main effect of TMS, we averaged across startle type and conducted three post hoc *t*-tests. Potentiated startle was significantly reduced in the active condition compared to both the sham (Active vs. Sham: *t*(18) = 2.38; *p* = 0.028) and the No TMS (Active vs. No TMS: *t*(18) = 2.31; *p* = 0.033) conditions, but no difference between the sham and the No TMS (Sham vs. No TMS: *t*(18) = 0.42; *p* = 0.677) conditions. Given that subjects were able to guess the active run at an above chance level, we combined subjects’ accuracy and confidence ratings into a continuous measure ranging from −1 (sure, but incorrect) to 1 (sure and correct), and included this in the original ANOVA. Importantly, the main effect of TMS was still significant (*f*(2,34) = 3.489; *p* = 0.042) and there were no significant main effects or interactions with our accuracy/confidence measure (*p*s > 0.05). Similarly, if we rerun the Active vs. Sham, Active vs. No TMS, and Sham vs. No TMS t-tests above as repeated-measures ANOVAs with accuracy/confidence as a covariate, we still observe significant main effects for the Active vs. Sham (*f*(1,17 = 8.376; *p* = 0.010) and Active vs. No TMS (*f*(1,17 = 4.569; *p* = 0.047) comparisons, and no main effect for the Sham vs. No TMS (*f*(1,17 = 0.011; *p* = 0.918) comparison. In addition, we found no significant main effects or interactions with accuracy/confidence in any of the ANOVAs (*p*s > 0.05). In addition, to test for laterality effects, we re-ran the original ANVOA adding hemisphere as a between-subjects measure, and found no significant main effects or interactions with this variable (all *p*s > 0.05).

### Ratings

As with startle, we quantified fear and anxiety from the online ratings, using the same equations described above (Fear: P cue – P ITI; Anxiety U ITI – N ITI). We then performed a 2 (Startle type: FPS vs. APS) × 3 (Stimulation type: Active, vs. Sham, vs. No TMS) repeated measures ANOVA on the results. As with startle, we found a significant main effect of fear vs. anxiety (*f*(1,18) = 37.85; *p* < 0.001). However, this effect was in the opposite direction with ratings being higher for anxiety compared to fear. In contrast, we found no main effect for TMS type (*f*(2,36) = 0.64; *p* = 0.532) and no interaction (*f*(2,36) = 1.44; *p* = 0.251).

## Discussion

We administered online 1-Hz rTMS to the parietal cortex during the NPU threat task. We measured fear and anxiety through potentiated startle responses and concurrent on-screen anxiety ratings. We found that rTMS reduced both fear- and anxiety-potentiated startle, but did not affect the online anxiety ratings. We also found that these results were unaffected by the laterality of the stimulation, or the individual’s ability to distinguish between active and sham stimulation. Together these results suggest that the parietal cortex plays a causal role in the elevated arousal that mediates potentiated startle responses, and that inhibiting activity using low-frequency rTMS is sufficient to reduce physiological arousal associated with fear and anxiety during threat. They also have implications for the use of noninvasive neuromodulation for the treatment of anxiety disorders. These points will be addressed below.

It is known that anxiety can impact attention control^26^, and that anxiety patients have trouble focusing attention^27,28^. This manifests in deficits in a variety of cognitive tasks, including working memory^29–31^. Importantly, the parietal cortex is critical for endogenous shifts in attention^32–34^. It also receives top-down feedback from the dlPFC during working memory manipulation^35^, playing a critical role in the maintenance of structured information^36^. This coordinated frontoparietal activity, in conjunction with the default mode network, has also been associated with the maintenance of internal thought^37^. Together these results suggest that the parietal cortex may be involved with endogenous shifts in attention toward threat during the NPU task. Therefore, by inhibiting parietal cortex activity during the NPU task, it is possible that we were reducing subjects’ tendency to shift their attention toward the shock threat, thereby reducing their threat-related anxiety.

This threat-related hypervigilance is a prominent symptom of clinical anxiety, cutting across multiple diagnoses^2^, and these results suggest that it may be mediated by a hyperactive parietal cortex^33,34,36,38,39^. This diminished attention control may explain why (1) individuals with specific phobias may show attentional biases to threatening information^40–43^, (2) generalized anxiety disorder (GAD) patients have difficulties maintaining attentional focus^44–47^ anxiety interferes with the manipulation of items in working memory^29^. If this is the case, then inhibitory parietal rTMS should also reduce the attention bias to threat seen in anxiety patients. Furthermore, we would predict that this rTMS protocol could be combined with conventional attention bias modification therapies to boost their efficacy.

Low-frequency rTMS of right parietal cortex leads to decreases in depressed mood and attentional bias to fearful faces^48^, and extended treatment with low-frequency right parietal stimulation leads to better emotion expression recognition in depressed patients^49^. However, it should be noted that extended treatment with this stimulation protocol did not lead to a significant reduction in depression symptoms in a recent clinical trial^50^. Future research needs to be conducted to explore the suitability of parietal inhibition for anxiety reduction. What does seem clear is that rTMS to posterior parietal regions affects attentional processes, particularly orienting. For instance, on recent study showed that stimulation of the right dorsal posterior parietal cortex resulted in enhancement in cued target detection when images are presented to the right hemifield^51^. These results are consistent with the hypervigilance hypothesis stated above, suggesting that targeting parietal regions during periods of elevated anxiety may reduce the hypervigilance experienced by anxiety patients, and may reduce attentional biases to threat stimuli.

One surprising finding in the current study is that we observed reductions in potentiated startle, but not anxiety ratings. Indeed, we generally expect a high correspondence between startle and ratings across experimental conditions. However, there is a precedent for dissociations between physiological and psychological expressions of fear and anxiety^52–55^. At a more fundamental level, we understand that anxiety is multifaceted, engaging multiple neural networks^56^, and encompassing a variety of distinct symptom domains^2^. The current manipulation, threat of predictable and unpredictable shock, can induce robust increases in physiological arousal^15,16^, impairments in performance on tasks that require working memory processes^57,58^, and improvements in tasks that require sustained attention^59^. Accordingly, it is clear that instructed threat impacts behavior across multiple domains important for anxiety symptomatology^60^. However, the benefit of TMS as an intervention is that one can potentially selectively target distinct symptom domains at the individual level. More work is definitely needed, but parietal inhibition may propose promise as a future treatment in its own right, or as an add-on to existing treatments.

Although our current results suggest a promising role for parietal inhibition in the treatment of anxiety, the majority of the anxiety-related rTMS research is focused on prefrontal stimulation. In general, researchers tend to manipulate the site of stimulation (left vs. right) and the frequency of stimulation (low-frequency vs. high frequency). High frequency left dlPFC stimulation and low-frequency right dlPFC stimulation tend to enhance anxiety regulation^61–67^, while high-frequency stimulation to the right dlPFC tends to enhance anxiety expression^68–70^. However, this distinction is not universal^71–73^. Consistent with this laterality effect, we recently measured fear- and anxiety-potentiated startle before and after 10 Hz stimulation to the right dlPFC, and found that 10 Hz stimulation increased anxiety-potentiated startle, suggesting that the right dlPFC may be important for anxiety expression rather than regulation^17^.

Importantly, frontoparietal interactions may be important for treatment response. Connectivity based parcellation shows that the IPS is strongly connected to prefrontal regions important for attentional processes^74^. Indeed, our previous results suggest that this region may be a cortical connectivity hub^14^. Together these results suggest that targeting parietal cortex may impact frontal circuits, and vice versa. Consistent with this hypothesis, stimulation to the IPS, which shows strong prefrontal connections, lead to impairments on a stop signal task compared to stimulation of the tempoparietal junction, an area not connected to the prefrontal cortex^74^. Also consistent with this network hypothesis of TMS effects, recent work has shown that prefrontal stimulation alters parietal activity^75^, and that the therapeutic effects of prefrontal stimulation may be mediated in part through these changes in parietal activity^76^.

There is increased interest in applications of noninvasive neuromodulation for the treatment of psychiatric disorders since rTMS was approved by the FDA for the treatment of depression^7^. Although there have been several clinical trials exploring the application of rTMS for the treatment of anxiety both as a symptom and as a disorder^66,77–79^, these clinical trials were largely based on the stimulation protocols developed for depression. These protocols, which largely rely on frontal stimulation^80–84^, are based on observations of asymmetric cortical EEG responses in depressed individuals^85,86^, and may not be optimally designed for the treatment of anxiety^87^. They also have the added limitation that they are often unpleasant or painful for patients, and may lead to more anxiety acutely^88^.

This research is a potential first step in the development of an anxiety-specific rTMS treatment protocol. We targeted our stimulation to a parietal region shown in previous neuroimaging work to be specifically involved in anxiety expression^14^. We used the combination of shock threat as a manipulation and potentiated startle as an outcome measure. Threat effects have been shown to be reliable both within session and across sessions^89–91^. Likewise, startle responses have also been shown to track well with clinical symptoms in pharmacological intervention studies, demonstrating external validity^89,91–93^. Finally, given that there are fewer peripheral off-target effects with parietal over frontal stimulation, this work also has the added benefit of greater tolerability, which may impact future treatment adherence. Together with the current rTMS results, these points suggest that the rTMS protocol used in this study has the potential to be an effective treatment for clinical anxiety, necessitating a great deal of additional research.

### Future directions

In this work, we discovered a novel application of rTMS that reduced threat-related anxiety in healthy volunteers. As mentioned above, these results have the potential to inform novel neuromodulatory treatments of clinical anxiety, however, the pathway to this aim will require several additional studies using both healthy volunteers, and ultimately patients. As a next step, it will be important to identify the mechanism of this anxiety reduction using simultaneous rTMS-fMRI, which will provide information about the downstream effects of parietal rTMS. Next, it will be important to determine the extent to which 1 Hz rTMS to the parietal cortex can induce long-lasting changes in parietal activity. For this, it will be important to conduct multiple neuromodulation sessions to generate a cumulative effect of the stimulation, and then test the effects in a subsequent post-neuromodulation test session. In addition, there should be sufficient time between the last neuromodulation session and the test to ensure that the acute effects of the rTMS are no longer active. Finally, it will be important to test this protocol in a large-scale clinical trial with generalized anxiety disorder patients.

### Strengths and limitations

There were several strengths to the current study. First, we used a reliable and validated approach to induce and measure anxiety in healthy volunteers. Second, we targeted a neurophysiological process engaged by acute anxiety, supported by evidence from multiple neuroimaging modalities. Third, we targeted this process at the individual level using a novel application of global brain connectivity to identify the local parietal connectivity hub. Finally, we optimized the stimulation by using e-field modelling to determine the optimal coil orientation for each subject, and tracked the position/orientation of the coil in real time using online neuronavigation.

Weaknesses should also be considered. First, subjects were able to distinguish between active and sham stimulation at an above chance level. Adequate blinding in rTMS studies is critical, and ensuring that the sensation is similar between active and sham stimulation is one approach to ensuring blinding. In our study we attempted to deliver an electric stimulus simultaneous to the TMS pulse in the sham condition (electrodes were attached in both active and sham). However, we used vinyl stickypad electrodes, because thicker electrodes would interfere with stimulation, and it was difficult to get a reliable connection with the scalp using these. Future studies might use low-profile EEG electrodes held in place under a swim cap to decrease the impedance. Because of this, it is important to consider whether the current results were due to subjects’ expectations. Given that (1) there was no effect of active stimulation on concurrent anxiety ratings, and (2) there was no effect of subjects’ perception of active/sham order on potentiated startle responses, our results suggest that the finding of reduced potentiated startle during the active stimulation was not due to subject demand characteristics. Another limitation of the current study is the relatively low sample size (*N* = 19); however, we used a within-subject design and observed a medium to large effect size that was consistent for both fear- and anxiety-potentiated startle. It is important to note that effect sizes can be overestimated with such small samples, and future studies should increase their sample size to ensure an accurate estimation of the true effects. As additional, clinical research using this stimulation protocol is conducted, it will be important to increase the sample size of the studies to a level more appropriate for a clinical trial. In the meantime, it should be noted that we used a within subject active/sham/control comparison to maximize power, and counterbalanced the order to minimize the carryover effects.

## Conclusions

This study found that low-frequency rTMS to the parietal cortex reduced fear and anxiety, as measured with startle. As a result, we believe that this stimulation protocol has promise in the development of a potential treatment for anxiety disorders. As a potential treatment, it has at least two advantages over current rTMS treatments. First, it is being developed specifically for anxiety. Second, there are fewer unpleasant off-target effects compared to frontal stimulation. Future clinical trials should be conducted to ensure that this stimulation protocol can affect long-lasting changes in symptoms experienced by anxiety patients.
